# Initial development and validation of a mitochondrial disease quality of life scale

**DOI:** 10.1016/j.nmd.2012.12.012

**Published:** 2013-04

**Authors:** J.L. Elson, M. Cadogan, S. Apabhai, R.G. Whittaker, A. Phillips, M.I. Trennell, R. Horvath, R.W. Taylor, R. McFarland, E. McColl, D.M. Turnbull, G.S. Gorman

**Affiliations:** a Mitochondrial Research Group and NIHR Biomedical Research Centre for Ageing and Age-Related Disease, Institute for Ageing and Health, Newcastle University, Newcastle upon Tyne NE2 4HH, UK; b Institute of Genetic Medicine, Newcastle University, Newcastle upon Tyne NE1 3BZ, UK; c Institute of Cellular Medicine, Newcastle University, Newcastle NE1 7RU, UK; d Institute of Health and Society, Newcastle Clinical Trials Unit, Newcastle University, Newcastle NE2 4HH, UK; e Centre for Human Metabonomics, North-West University, Potchefstroom, South Africa

**Keywords:** Quality of life, Mitochondrial disease, Questionnaire

## Abstract

Mitochondrial diseases are a clinically diverse group of genetic disorders that often present to neurologists. Health related quality of life (HRQOL) is increasingly recognised as a fundamental patient based outcome measure in both clinical intervention and research. Generic outcome measures have been extensively validated to assess HRQOL across populations and different disease states. However, due to their inclusive construct, it is acknowledged that not all relevant aspects of a specific illness may be captured. Hence there is a need to develop disease specific HRQOL measures that centre on symptoms characteristic of a specific disease or condition and their impact. This study presents the initial conceptualisation, development and preliminary psychometric assessment (validity and reliability) of a mitochondrial disease specific HRQOL measure (Newcastle Mitochondrial Quality of life measure (NMQ)). NMQ is a valuable assessment tool and consists of 63 items within 16 unidimensional domains, each demonstrating good internal reliability (Cronbach’s *α* ⩾ 0.83) and construct validity.

## 1 Introduction

Mitochondrial disorders are a clinically multifarious group of genetic disorders that affect the central nervous system and skeletal muscles and other organs heavily dependent on aerobic metabolism. They are typically characterised by multi-system involvement. They have extensive phenotypic and disease burden variability and although a disease rating scale [1] exists which monitors the spectrum and rate of progression of disease, it does not assess the psychological and social impact of having a mitochondrial disorder.

Health related quality of life (HRQOL) is increasingly recognised as a fundamental patient-based outcome measure in both clinical intervention and research. Generic outcome measures have been extensively validated to assess HRQOL across populations and different disease states. However due to their inherent generic construct they may not fully capture all relevant aspects of a specific illness [2]. It is recognised that there is a need to develop disease-specific HRQOL measures that centre on the symptoms and impact characteristic of a specific disease or condition [3]. We present the initial conceptualisation, development and preliminary psychometric evaluation (validity and reliability) of a mitochondrial disease-specific HRQOL measure.

## 2 Methods

### 2.1 Subjects and source of items

Eligible participants were defined as adult patients (18 years and above) with a known biochemical or genetic diagnosis of mitochondrial disease. Subjects were excluded if they had cognitive impairment that prevented questionnaire comprehension or were unable to read English.

Domain and item content validity of the pilot questionnaire was assured by deriving item content through semi-structured key-informant interviews. Investigators conducted two focus groups to explore patients’ perceptions of the physical, psychological and social impact of mitochondrial disease through open questions and the employment of the ‘Gap Model’ technique [4]. Themes which were perceived as influential on HRQOL in mitochondrial disease and arose during the interview processes or from review of other neurological and HRQOL instruments [5–8] were categorised into representative life domains as verified independently by three investigators.

### 2.2 Questionnaire design

Questionnaire design was determined by reference to questionnaire design guidelines and by advice from a survey methods consultant. A four week recall period and an adjective rating scale (never, occasionally, sometimes, often, always, not applicable) were selected as the most appropriate recall time-frame and response scale respectively. To test basic comprehension and acceptability, and prior formal piloting, a first draft questionnaire was piloted on randomly selected patients with mitochondrial disease. Relevant changes were made.

### 2.3 Item reduction and validation

Patients attending the Newcastle mitochondrial disease clinic were asked to complete the pilot questionnaire to (1) confirm that the items and domains selected from the interviews and review of other HRQOL instruments were representative; (2) to highlight any issues that may have been omitted; (3) to facilitate item reduction. Preliminary data were evaluated to assess endorsement rate, scale reliability and variability. With the use of five criteria, both item and domain contributions to the scale were evaluated. Domains were assessed by prespecified criteria [9]: (1) Items as a whole were evaluated using frequency of endorsement. Those with very high (>80%) or low (<20%) endorsement rates, of any one category, were removed as such items are unlikely to be sufficiently discriminatory. (2) Domain variability was assessed using factor analysis. An Eigen value cut off point of 0.95 was used, as it was a requirement that each domain would be unidimensional. Any items within a domain with a cumulative Eigen value >0.95 were eliminated. (3) To test the internal reliability of each item within a domain, item-total correlations were calculated; an item-total correlation of greater than 0.20 were accepted as indicative of adequate internal reliability; items with item-total correlation of ⩽0.20 were eliminated. (4) Items were removed where the Cronbach’s *α* for the constituent domain was greater if that item was removed than if the item was retained. (5) Domains with a Cronbach’s *α* <0.70 or >0.95 were dropped. Once redundant items were removed face to face cognitive interviews were conducted to verify comprehension and ensure face validity.

### 2.4 Psychometric evaluation

The amended questionnaire (NMQ: Newcastle Mitochondrial-Quality of life measure) was piloted again to facilitate further content validity and psychometric evaluation. Subjects were asked to complete NMQ and a validated HRQOL measure (SF-36) [10]. Data were evaluated to assess endorsement rate and scale reliability (internal consistency reliability) and variability. Construct validity was assessed by comparing questionnaire responses to comparable elements of the SF-36. Multi-trait analysis was used to examine correlations within and across similar and dissimilar domains in each instrument; we anticipated that scales measuring similar constructs (for example, Role physical (SF-36) and mobility (NMQ)) would be more highly correlated with one another than those tapping dissimilar constructs. Known group validity was established using the Newcastle Mitochondrial Disease Adult Scale (NMDAS) [1], a validated measure of disease burden and a surrogate for phenotypic severity, with one-way analysis of variance performed. Subjects were divided into 3 groups according to their NMDAS scores (Group 1: 0–24 (mild); Group 2: 25–49 (moderate); Group 3: 50 and above (severe)) disease burden. This allowed the assessment of how well the questionnaire was able to distinguish changes in quality of life in relation to disease severity. It was expected that there would be a negative relationship between NMQ and NMDAS scores, that is, the greater the disease burden, the poorer the perceived quality of life, reflected in lower NMQ scores.

### 2.5 Scoring methods

The raw score (obtained by adding across all items in the domain) for each NMQ domain (Never: 5; rarely: 4, sometimes: 3, often: 2 and always: 1) was transformed to a 0–100 score, as in the calculation of subscale scores for the SF-36, by the following formula

[Totaldomainscore-thenumberofitems]/[maximumpossible-minimumpossiblescoreperdomain]×100

### 2.6 Statistical analysis

All statistical analyses were performed using Minitab version 17.

## 3 Results

### 3.1 Item generation, reduction and validation

**Fig. 1:**
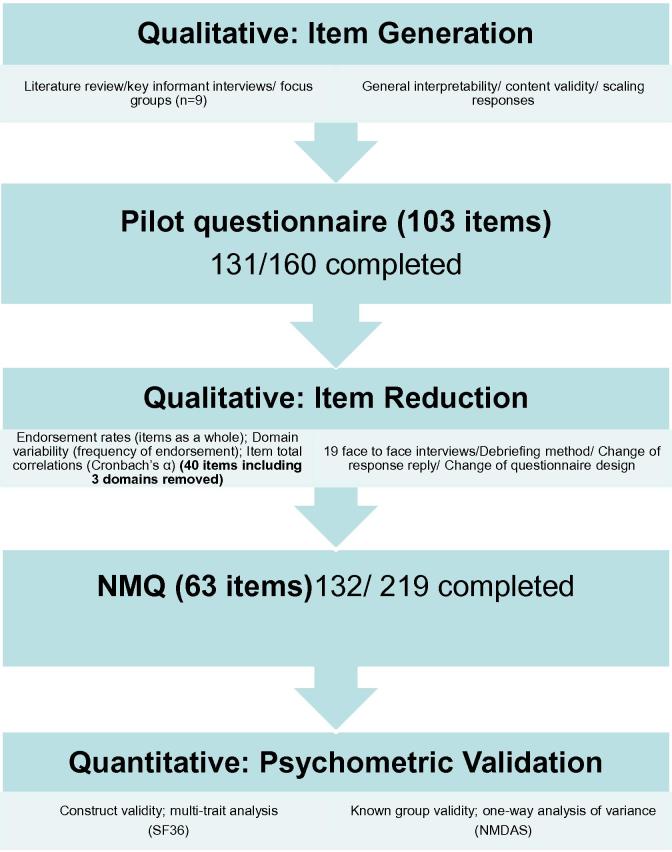
Summary flow chart of the steps involved in the derivation of NMQ.

Derivation of NMQ is summarised in Fig. 1. Nine patients (5 men; mean age 41 years (SD 17 years)) attended the semi-structured key-informant interviews. Six randomly selected patients with mitochondrial disease (3 men; age range 28–56 years) completed the first draft questionnaire. From this, a pilot questionnaire consisting of 19 domains (103 items) as determined by the research team covering all themes mentioned during patient interviews and named according to their constituent items, was generated (Fig. e-1). One hundred and sixty patients were invited to complete the pilot questionnaire; 131 were returned (response rate of 81.9%).

Face to face cognitive interviews were conducted to check on face and content validity and how understandable the items were. Nineteen patients were interviewed (8 men; mean age 41 years (SD 14 years)). Amongst the issues raised and subsequently addressed were: (1) font size was increased to 14 for ease of readability; reflecting how visual problems are common in mitochondrial cytopathies (2) the response scale was adjusted: ‘not applicable’ was omitted; and ‘occasionally’ was changed to ‘rarely’. (3) Questionnaire layout was changed to incorporate a colour–contrast background with individual response boxes and clearer instructions for ease of completion. Factor analysis showed that all domains were unidimensional, that is, measured one aspect of disease in both the pilot and final NMQ questionnaires. The internal reliability of retained domains remained high in all domains (Cronbach’s *α* >0.83) (Table 1). Item and domain contributions to the scale, according to the pre-specified criteria were also evaluated. These processes resulted in the removal of 40 items, including 3 whole domains: stroke, seizures and work due to low endorsement (Fig. e-2a and b), cumulative Eigen values >0.95 (Table e-1) and/or Cronbach’s *α* >0.95 (Table 1), as well as the category of ‘not applicable’ from the response scale. The final questionnaire (NMQ) consisted of 63 items within 16 unidimensional domains (Fig. e-3).

### 3.2 Psychometric evaluation – construct and group validity

Two hundred and nineteen patients were asked to complete NMQ and SF-36; 132 were returned (response rate: 61.3%). Respondents comprised 91 women (mean age 53 years, SD 14) and 41 men (mean age 48, SD 16). Sixty-three respondents had a diagnosed point mutation (m.3243A>G MELAS mutation (mitochondrial encephalopathy, lactic acidosis and stroke-like episodes) (39); m.8344A>G MERRF mutation (myoclonic epilepsy with ragged red fibres) (9); LHON (Leber’s hereditary optic neuropathy) (2) NARP (neuropathy, ataxia and retinitis pigmentosa) (1); other mitochondrial tRNA (mt-tRNA) mutations (12). Forty-three participants were diagnosed with multiple mtDNA deletions (unspecified nuclear genetic defect (26), OPA1 (optic atrophy type 1) (3), POLG1 (polymerase gamma) (6) and PEO1 (Twinkle) (8)). Twenty-six participants were diagnosed with heteroplasmic single-large scale mtDNA deletion. Salient clinical features of respondents as a whole are summarised in Table e-2. With respects to the m.3243A>G point mutation group, 13 patients had a history of current or remote seizures and strokes, 14 subjects had either a classical maternally inherited diabetes and deafness (MIDD) or isolated sensori neural hearing loss (SNHL) phenotype, 10 had a pure myopathic phenotype and 2 respondents were asymptomatic carriers.

NMQ domains correlated with related SF-36 domains confirming good construct validity (Table 2). There were no comparable domains for vision, food and digestion, memory and diabetes. No systematic differences in NMQ domain scores were found with respect to age (Pearson’s product-moment correlation co-efficient), gender (independent-samples *t*-test) or genotype (One-way analysis of variance) (Table e-3). Known group validity analysis revealed that NMQ subscale scores varied with disease severity in all domains except emotional well being, stigma and diabetes (Table 3).

## 4 Discussion

Mitochondrial disease can have a significant impact on patients’ quality of life. The high response rates of both pilot and NMQ questionnaires provide evidence that patients with mitochondrial disease welcomed the opportunity to report on their quality of life. The breadth of themes volunteered by patients during NMQ derivation reflects the large spectrum of symptoms in patients with mitochondrial disease and their wide-ranging influence on many aspects of functional and mental health and well being.

HRQOL is important for understanding the impact and progression of chronic disease. Indeed, in a condition were the natural history of the disease is poorly understood and therapeutic options are limited, long-term preservation of HRQOL in mitochondrial disease poses a real challenge.

SF-36 and its abbreviated version SF-12 are currently the only tools used routinely for measuring patient-reported outcomes in our patients with mitochondrial disease. Stroke, seizures and work domains of the initial pilot questionnaire did not show adequate construct validity or internal consistency reliability and were excluded from NMQ. Criticism in the past has been that the structure and content of HRQOL measures do not capture all of the issues relevant to the patient and often do not allow them to indicate the impact of disease burden on their perceived QOL. We endeavoured, at all stages of the construct of NMQ to correct this shortcoming during its conceptualisation and validation. Although our initial bias was to include domains that we felt from a physician-centred perspective were relevant to patient’s quality of life such as stroke and seizures; repeatedly these aspects were not considered important to this patient group as a whole. This may reflect the low prevalence of strokes (12%) and seizures (14%) within our cohort and the genotypic-phenotypic specificity of such symptoms. However, this may also suggest that the domain items included in the original pilot questionnaire lacked ability to discriminate the impact of seizures and stroke on perceived quality of life within the affected group of patients.

In an attempt to address two of the shortcomings of the SF-36, NMQ includes domains of cognitive functioning (memory/cognition) and sexual functioning (personal relationships). Cognitive function domain originally consisted of four items and was reduced to one by combining two items (‘found it difficult to make decisions’ and ‘have you felt your thinking is confused’) for ease of completion. Two further items (‘had problems with memory’ and ‘had problems with your concentration’) were omitted due to repeatedly low endorsement rates. No amendments were made to the personal relationships domain; the key-informant interviewees highlighted this domain as an important quality of life measure not routinely assessed; which the group as a whole endorses. Impact of mitochondrial disease on work and employment are addressed within several subscales. Other life domains encompassed in NMQ but not SF-36 includes vision, memory, food and digestion and diabetes. Within specific domains, other items not routinely assessed in generic HRQOL and pertinent to patients’ with mitochondrial disease have been addressed such as hearing loss (communication domain) which is recognised to be prevalent in mitochondrial disease.

Completion of NMQ is simple and quick (on average 3–5 min) and scoring of the questionnaire is similar to that of the SF-36. Each domain is transformed into a 0–100 scale on the assumption that each question carries equal weight, thus the higher the percentage NMQ domain score the greater the perceived quality of life and health status.

The psychometric evaluation of NMQ provides good evidence of both reliability and validity. Internal consistency of each of the domains exceeded the 0.7 threshold and item-total correlations exceeded 0.2 for all items. NMQ domain scores of mobility, muscle stiffness, emotion, family role and social role show moderate to strong correlation between similar SF-36 domains (*r* values 0.3–0.69), with the strongest correlation occurring with NMQ personal relationships’ domain and SF-36 role physical domain. NMQ domains of activities of daily living, energy, communication, pain, migraine and stigma show poor correlation with their comparative SF-36 health concepts. These domains remained within NMQ as they were considered important by our patient interview group and investigators during content validity assessment. We acknowledge that NMQ and SF-36 domain pairing for statistical analyses may not have been ideal, for example, migraine and body pain, and communication and social functioning but it is also recognised that internal consistency is affected by the degree of item correlation and the number of items within a domain with considerable variation between NMQ and SF-36 domain item content noted.

These findings are encouraging with further psychometric assessment and revision of NMQ required particularly in relation to the domains of stroke and seizures. Test-retest reliability and responsiveness to change will be undertaken in the future. This maybe best facilitated by a European multi-centre follow-up analysis. With little known about progression of mitochondrial disease, assessing HRQOL may contribute to our understanding of disease impact and evolution over time. Indeed, if HRQOL is a true marker of disease burden and better reflects functional status and well-being than traditional biological outcome measures, then it is imperative that we have a validated disease-specific tool which will facilitate longitudinal time-based monitoring in research and clinical practise.

## Disclaimers

M.C.: Nothing to declare. J.L.E. receives support from a Research Council UK RCUK Academic fellowship (JLE). S.A.: Nothing to declare. R.W.: Nothing to declare. A.P. Nothing to declare. M.I.T is supported by a fellowship from Diabetes UK. R.H. receives research support from the Newcastle upon Tyne Hospitals NHS Charity, and the Academy of Medical Sciences (UK). R.M.F. is supported by a HEFCE/DoH Clinical Senior Lecturer Award and the MRC Centre for Translational Research in Neuromuscular Disease Mitochondrial Disease patient Cohort (UK) (G0800674). E.M.C. is part supported by the MRC Centre for Neuromuscular Diseases (G0601943). D.M.T. is supported Wellcome Trust Centre for Mitochondrial Research (906919), the MRC Centre for Neuromuscular Diseases (G0601943), MRC Centre for Translational Research in Neuromuscular Disease Mitochondrial Disease Patient Cohort (UK) (G0800674), Newcastle University Centre for Brain Ageing and Vitality supported by BBSRC, EPSRC, ESRC and MRC (G0700718). R.W.T. is supported by a Wellcome Trust Programme Grant (074454/Z/04/Z), Wellcome Trust Centre for Mitochondrial Research (906919) and the MRC Centre for Translational Research in Neuromuscular Disease Mitochondrial Disease Patient Cohort (UK) (G0800674). G.S.G. is funded by the UK NIHR Biomedical Research Centre for Ageing and Age-related disease award to the Newcastle upon Tyne Foundation Hospitals NHS Trust.

## Figures and Tables

**Supplementary Fig. e-1:**
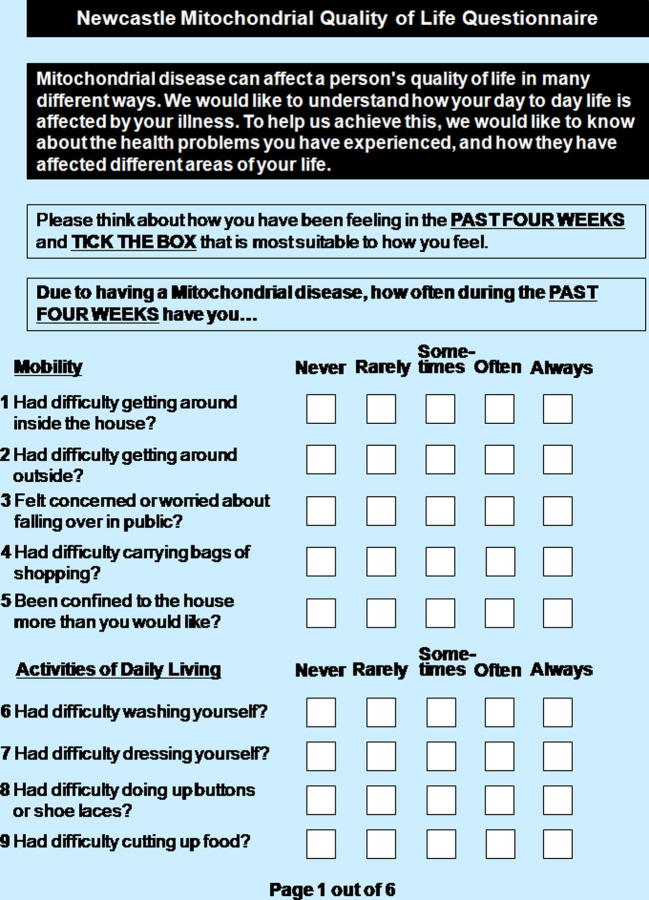
A copy of the original pilot questionnaire, consisting of 19 domains (103 questions), as determined by the research team covering all themes mentioned during patient interviews and named accordingly to their constituent items.: Page 1 out of 6

**Supplementary Fig. e-1:**
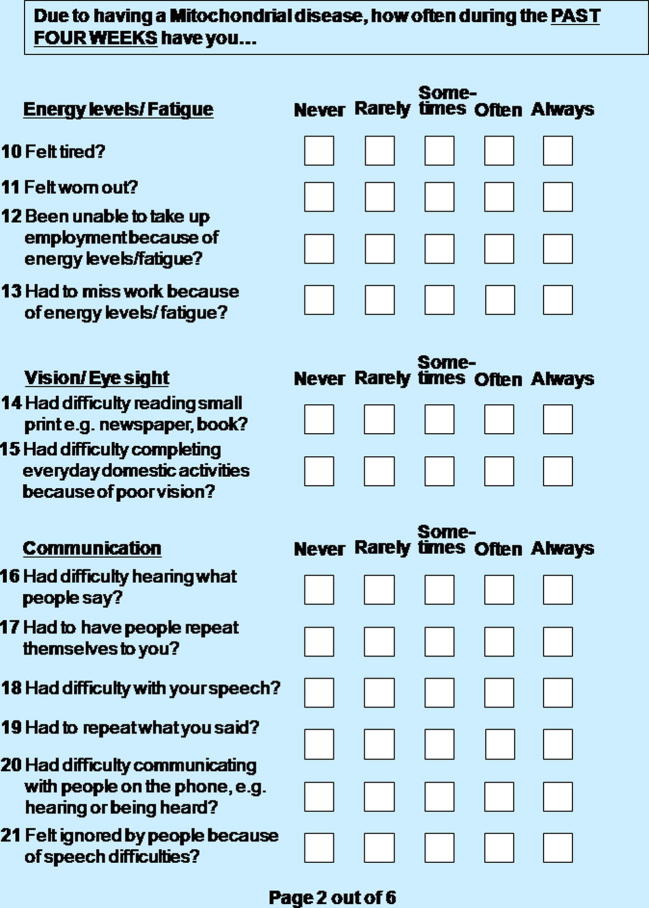
Page 2 out of 6

**Supplementary Fig. e-1:**
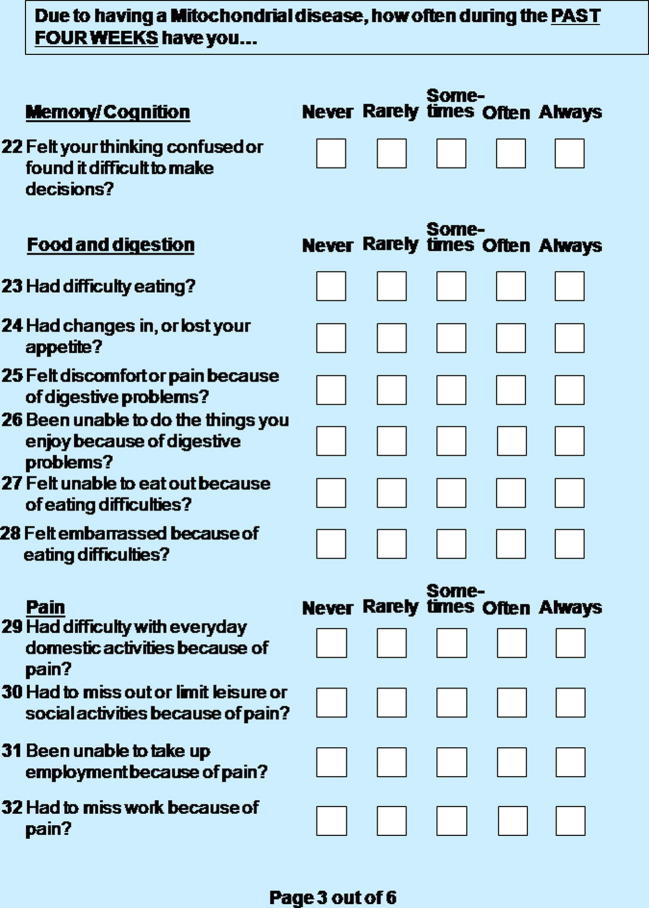
Page 3 out of 6

**Supplementary Fig. e-1:**
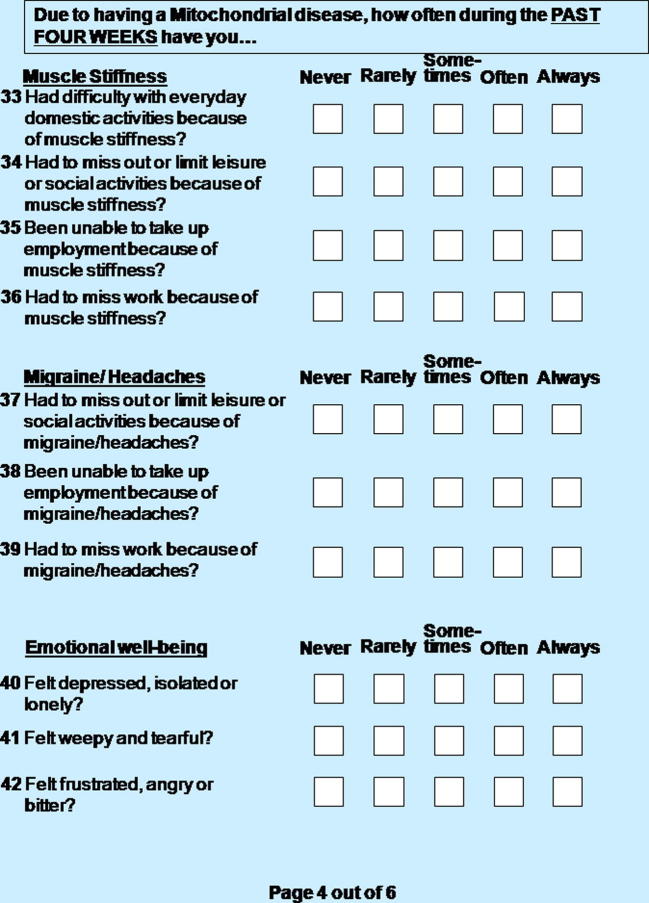
Page 4 out of 6

**Supplementary Fig. e-1:**
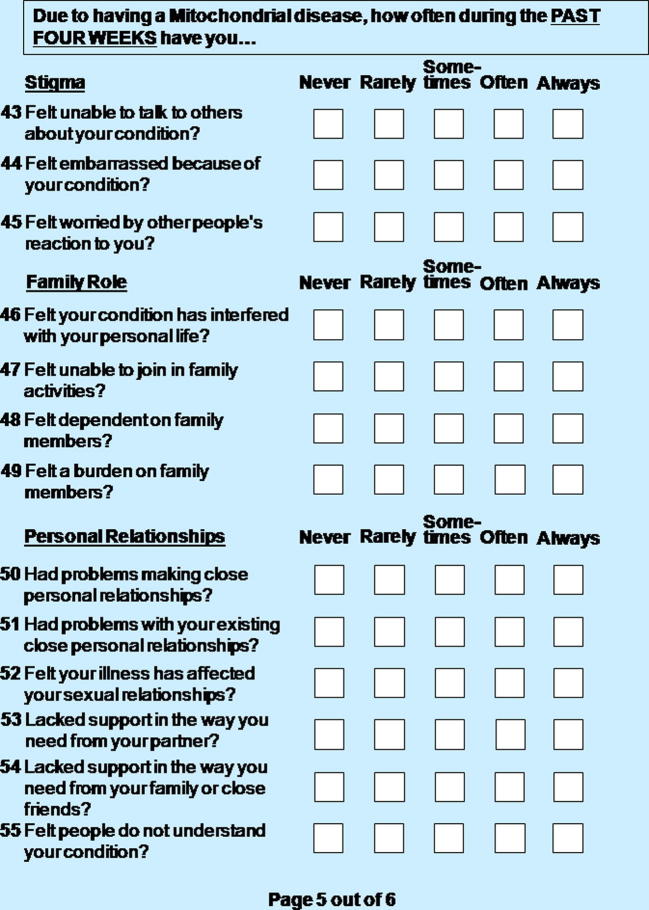
Page 5 out of 6

**Supplementary Fig. e-1:**
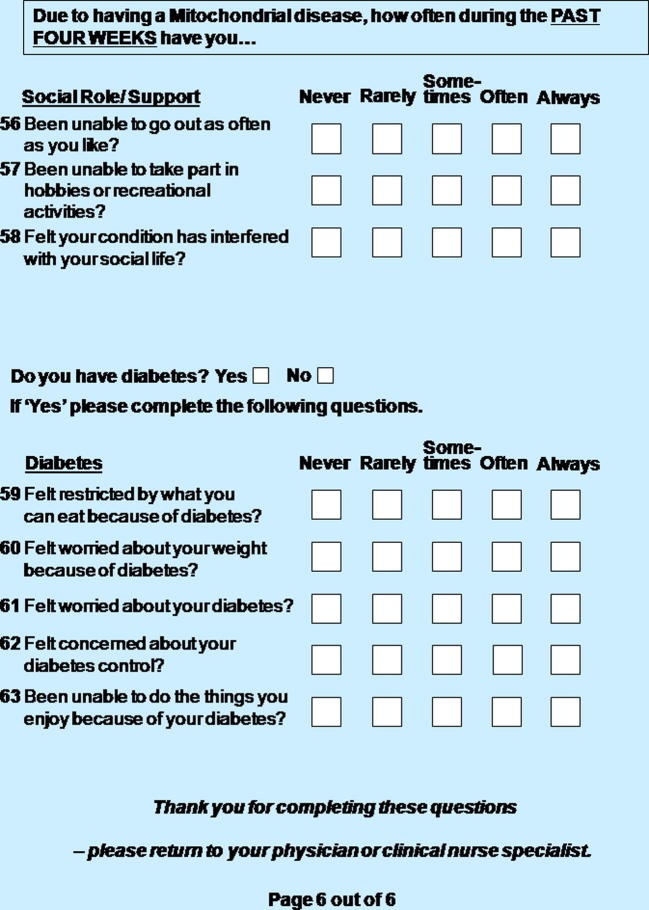
Page 6 out of 6

**Table 1:** A summary of the internal reliability of domains within the initial draft and final version of the mitochondrial disease specific quality of life questionnaire (NMQ) as assessed by Cronbach’s *α* demonstrating good internal reliability.

NMQ domain	No. of items (1st draft)	Cronbach’s alpha (1st draft)	No. of items (NMQ)	Cronbach’s alpha (NMQ)
Mobility	6	0.78	5	0.91
Activities of daily living	6	0.92	4	0.88
Vision	3	0.74	2	0.83
Memory	1	N/A	1	N/A
Pain	4	0.90	4	0.91
Migraine	4	0.82	3	0.89
Emotion	8	0.94	3	0.88
Stigma	6	0.92	3	0.88
Family role	4	0.90	4	0.87
Personal relationships	6	0.76	6	0.84
Social role	4	0.92	3	0.92
Diabetes	8	0.92	5	0.93
Stroke	5	1.0	N/A	N/A
Seizures	8	0.97	N/A	N/A
Work	3	0.94	N/A	N/A

N/A: not applicable.

**Table 2:** A summary of the construct validity of NMQ domains compared to equivalent SF-36 domains as assessed by Pearson’s correlation co-efficient showing good construct validity.

NMQ domains	SF-36 domain						
	PF	RP	VT	SF	BP	RE	MH
Mobility	*0.4*	*0.37*					
ADL	0.26	0.18					
Energy			0.04				
Communication				0.22			
Pain					0.26		
Muscle stiffness					*0.52*		
Migraine					0.20		
Emotion						0.23	*0.32*
Stigma							0.20
Family role		0.24		*0.36*		0.24	
PR		**0.73**		0.24		0.20	
Social role		0.29		*0.30*		0.25	

PF = physical function; RP = role physical; VT = vitality; SF = social function; BP = bodily pain; RE: role emotion; MH = mental health; PR = personal relationships. Italics denotes moderate correlation (Pearson’s product moment correlation co-efficient (*r* = 0.3–0.69); Bold denotes strong correlation (*r* > 0.7).

**Table 3:** Known group validity analysis of NMQ domains as assessed by one-way analysis of variance showing that as disease severity increases, and NMQ domain scores decrease in all domains except stigma, emotional well-being and diabetes.

NMQ domains	*F*	*p*
Activities of daily living	21.02	0.0005
Mobility	21.63	0.0005
Energy levels/fatigue	11.92	0.0005
Communication	9.87	0.0005
Vision/eyesight	6.16	0.003
Memory/cognition	3.45	0.035
Food and digestion	5.17	0.007
Pain	13.23	0.0005
Muscle stiffness	11.28	0.0005
Migraine/headaches	7.31	0.001
Emotional well being	2.77	0.067
Stigma	1.15	0.319
Family role	5.10	0.007
Personal relationships	5.46	0.0005
Social role/support	15.88	0.0005
Diabetes	0.38	0.686

## Appendix A Supplementary data

Supplementary Fig. e-2 Bar charts show endorsement levels and variation in response to each domain item for the pilot (a) and NMQ (b) questionnaires.

Supplementary Fig. e-3 Newcastle Mitochondrial Quality of Life Questionnaire (NMQ) consisting of 63 items within 16 unidimensional domains: mobility, ADL, energy and fatigue, vision, communication, memory and cognition, food and digestion, pain, muscle stiffness, headaches and migraine, emotional well-being, stigma, family role, personal relationships, social role and support and diabetes.

Supplementary Table e-1 A table illustrating principle component analysis of the correlation matrix for the domains of seizures, stroke and work, showing that all items of the stroke domain, and all items except the first in both seizures and work domains have cumulative Eigen values over 0.95 and therefore do not add additional information to that domain.

Supplementary Table e-2 Salient clinical features of the 132 respondents to the NMQ questionnaire.

Supplementary Table e-3 A table illustrating that genotype (one-way analysis of variance) gender (independent *t*-test) and age (Pearson’s moment correlation co-efficient) have no impact on NMQ domain scores when statistical significance is set at *p* < 0.003 (Bonferroni correction).
