# Supplementary material for: Initial development and validation of a mitochondrial disease quality of life scale
Source: Neuromuscul Disord. 2013 Apr;23(4):324–9. doi: 10.1016/j.nmd.2012.12.012 (PMC3841574; doi:10.1016/j.nmd.2012.12.012)
Supplement: Supplementary Fig. e-2 — Bar charts show endorsement levels and variation in response to each domain item for the pilot (a) and NMQ (b) questionnaires. [file mmc1.docx]

# figure e2- a

Key

MOB = Mobility, ADL = Activities of Daily Living, EL&F = Energy levels and Fatigue, EYE = Vision and Eye sight, COM = Communication, M&C = Memory and Cognition, F&D = Food and digestion, Pain = Pain, Mus&St = Muscle Stiffness, Mig = Migraine and Headaches, EWB = Emotional well-being, F-role = Family Role, PR = Personal Relationships, S-role = Social Role and Support

# figure e2- b

Key

MOB = Mobility, ADL = Activities of Daily Living, EL&F = Energy levels and Fatigue, EYE = Vision and Eye sight, COM = Communication, M&C = Memory and Cognition, F&D = Food and digestion, Pain = Pain, Mus&St = Muscle Stiffness, Mig = Migraine and Headaches, EWB = Emotional well-being, F-role = Family Role, PR = Personal Relationships, S-role = Social Role and Support
