# Supplementary material for: Initial development and validation of a mitochondrial disease quality of life scale
Source: Neuromuscul Disord. 2013 Apr;23(4):324–9. doi: 10.1016/j.nmd.2012.12.012 (PMC3841574; doi:10.1016/j.nmd.2012.12.012)
Supplement: Supplementary Fig. e-3 — Newcastle Mitochondrial Quality of Life Questionnaire (NMQ) consisting of 63 items within 16 unidimensional domains: mobility, ADL, energy and fatigue, vision, communication, memory and cognition, food and digestion, pain, muscle stiffness, headaches and migraine, emotional well-being, stigma, family role, personal relationships, social role and support and diabetes. [file mmc2.pdf]

NEWCASTLE MITOCHONDRIAL QUALITY OF LIFE QUESTIONNAIRE

Mitochondrial disease can affect a person's quality of life in many different ways. We would like to understand how your day to day life is affected by your illness. To help us achieve this, we would like to know about the health problems you have experienced, and how they have affected different areas of your life.

Please think about how you have been feeling in the past four weeks, and tick the box that is most suitable to how you feel. If someone helps you fill this questionnaire, please make sure they accurately reflect how you feel. Thank you.

Due to having a Mitochondrial disease, how often during the last four weeks have you ...

		Never	Occasi-onally	Some-times	Often	Always	Not applicable
	Mobility						
1	Had difficulty getting around inside the house?						
2	Had difficulty getting around outside?						
3	Needed someone else to accompany you when you went out?						
4	Felt frightened or worried about falling over in public?						
5	Had difficulty carrying bags of shopping?						
6	Been confined to the house more than you would like?						
	Activities of Daily Living						
7	Had difficulty washing yourself?						
8	Had difficulty dressing yourself?						
9	Had difficulty doing up buttons or shoe laces?						
10	Had difficulty cutting up food?						
11	Had difficulty completing housework?						
12	Had difficulty writing clearly?						
	Vision/ Eye sight						
13	Had difficulty reading a book?						
14	Had difficulty seeing the television well enough to enjoy a show?						
15	Had difficulty completing everyday activities?						
	Communication						
16	Had difficulty hearing what people say?						
17	Had to have people repeat themselves to you?						
18	Had difficulty with your speech?						
19	Had to repeat what you said?						
20	Had difficulty communicating with people on the phone, e.g. hearing or being heard?						
21	Felt ignored by people?						

Due to having a Mitochondrial disease, how often during the last four weeks have you ...

		Never	Occasi-onally	Some-times	Often	Always	Not applicable
	Energy levels/ Fatigue						
22	Felt full of energy?						
23	Felt tired?						
24	Felt worn out?						
25	Had difficulty with everyday domestic activities because of this, e.g. cooking, laundry?						
26	Been unable to do the things you enjoy because of this, e.g. hobbies?						
27	Been unable to take up employment because of this?						
28	Had to miss work because of this?						
	Food and digestion						
29	Had difficulty eating?						
30	Lost your appetite?						
31	Felt discomfort or pain because of digestive problems?						
32	Been unable to do the things you enjoy because of digestive problems?						
33	Felt unable to eat out because of this?						
34	Felt embarrassed because of this?						
	Diabetes						
35	Altered your diet?						
36	Felt restricted with your diet?						
37	Felt worried about your weight?						
38	Felt worried about your diabetes?						
39	Felt concerned about your diabetes control?						
40	Been unable to take up employment because of this?						
41	Had to miss work because of this?						
42	Been unable to do the things you enjoy because						
	Pain						
43	Had difficulty with everyday domestic activities because of pain, e.g. cooking, laundry?						
44	Had to miss out or limit leisure or social activities because of pain?						
45	Been unable to take up employment because of this?						
46	Had to miss work because of this?						
	Stiffness						
47	Had difficulty with everyday domestic activities because of pain, e.g. cooking, laundry?						
48	Had to miss out or limit leisure or social activities because of muscle stiffness?						
49	Been unable to take up employment because of this?						
50	Had to miss work because of this?						

Due to having a Mitochondrial disease, how often during the last four weeks have you...

		Never	Occasi-onally	Some-times	Often	Always	Not applicable
	Migraine						
51	Had difficulty with everyday domestic activities						
52	Had to miss out or limit leisure or social activities						
53	Been unable to take up employment because of						
54	Had to miss work because of a migraine?						
	Seizures						
55	Been worried about having another seizure?						
56	Been worried about hurting yourself during a seizure?						
57	Been worried about embarrassment or other social problems because of having seizures?						
58	Been worried about taking antiepileptic medication?						
59	Had difficulty with everyday domestic activities because of pain, e.g. cooking, laundry?						
60	Had to miss out or limit your leisure or social activities because of a seizure?						
61	Been unable to take up employment because of this?						
62	Had to miss work because of this?						
	Strokes						
63	Had to depend on someone else because of a stroke?						
64	Had difficulty with everyday domestic activities because of pain, e.g. cooking, laundry?						
65	Had to miss out or limit your leisure or social activities because of a stroke?						
66	Been unable to take up employment because of this?						
67	Had to miss work because of this?						
	Memory/ Cognition						
68	Found it difficult to make decisions?						
69	Have you felt your thinking is confused?						
70	Had problems with your memory?						
71	Had problems with your concentration?						
	Emotional well-being						
72	Felt depressed?						
73	Felt isolated and lonely?						
74	Felt weepy and tearful?						
75	Felt frustrated?						
76	Felt angry or bitter?						
77	Felt anxious?						
78	Felt worried?						
79	Felt not motivated to do things?						

Due to having a Mitochondrial disease, how often during the last <u>four</u> weeks have you...

	Stigma	Never	Occasi-onally	Some-times	Often	Always	Not applicable
80	Felt you had to conceal your condition from someone?						
81	Felt unable to talk to others about your condition?						
82	Felt embarrassed because of your condition?						
83	Felt embarrassed because of your appearance?						
84	Felt worried by other people's reaction to you?						
85	Avoided situations in public?						

	Personal Relationships	Never	Occasi-onally	Some-times	Often	Always	Not applicable
86	Had problems making close relationships?						
87	Had problems with your existing close personal relationships?						
88	Felt your illness has affected your sexual relationships?						
89	Lacked support in the way you need from your partner?						
90	Lacked support in the way you need from your family or close friends?						
91	Felt people do not understand your disease?						

	Family Role	Never	Occasi-onally	Some-times	Often	Always	Not applicable
92	Felt your condition has interfered with your personal life?						
93	Felt unable to join in family activities?						
94	Felt dependent on family members?						
95	Felt a burden on family members?						

	Social Role/ Support	Never	Occasi-onally	Some-times	Often	Always	Not applicable
96	Been unable to go out as often as you like?						
97	Been unable to take part in hobbies and recreational activities?						
98	Had to cancel meeting friends because of your condition?						
99	Felt your condition has interfered with your social life?						

	Work	Never	Occasi-onally	Some-times	Often	Always	Not applicable
100	Been unable to take up employment because of this?						
101	Had to miss work because of this?						
102	Felt your condition has affected your performance at work?						

	Overall	Excell-ent	Very good	Good	Fair	Poor
103	How would you describe your quality of life?					
