# Supplementary material for: Initial development and validation of a mitochondrial disease quality of life scale
Source: Neuromuscul Disord. 2013 Apr;23(4):324–9. doi: 10.1016/j.nmd.2012.12.012 (PMC3841574; doi:10.1016/j.nmd.2012.12.012)
Supplement: Supplementary Table e-1 — A table illustrating principle component analysis of the correlation matrix for the domains of seizures, stroke and work, showing that all items of the stroke domain, and all items except the first in both seizures and work domains have cumulative Eigen values over 0.95 and therefore do not add additional information to that domain. [file mmc3.docx]

table e-1

| **Question (pilot questionnaire)** | **Cumulative Eigen value** |
| --- | --- |
| **Seizures** | |
| 55 | 0.91 |
| 56 | 0.98 |
| 57 | 1.0 |
| 58 | 1.0 |
| 59 | 1.0 |
| 60 | 1.0 |
| 61 | 1.0 |
| 62 | 1.0 |
| **Stroke** | |
| 63 | 1.0 |
| 64 | 1.0 |
| 65 | 1.0 |
| 66 | 1.0 |
| 67 | 1.0 |
| **Work** | |
| 100 | 0.90 |
| 101 | 0.98 |
| 102 | 1.0 |
