# Supplementary material for: Initial development and validation of a mitochondrial disease quality of life scale
Source: Neuromuscul Disord. 2013 Apr;23(4):324–9. doi: 10.1016/j.nmd.2012.12.012 (PMC3841574; doi:10.1016/j.nmd.2012.12.012)
Supplement: Supplementary Table e-2 — Salient clinical features of the 132 respondents to the NMQ questionnaire. [file mmc4.docx]

table e-2

| Clinical feature | No. of patients | Percentage of all patients |
| --- | --- | --- |
| Hearing loss | 32 | 24 |
| Ataxia | 39 | 30 |
| Myopathy | 41 | 31 |
| Diabetes mellitus | 18 (4) | 14 |
| Ataxia | 39 | 30 |
| Stroke-like episodes | 16 | 12 |
| Seizures | 18 | 14 |
| Encephalopathy | 12 (3) | 9 |
| Ptosis/PEO | 65 | 49 |
| Neuropathy | 8 | 6 |
| Myoclonus | 8 | 6 |
| Cardiomyopathy | 3 | 2 |
| Ventricular pre-excitation | 3 | 2 |
| Lipomata | 6 | 4 |
| Parkinsonism/Dystonia | 4 | 3 |
| Visually Impaired | 7 | 5 |
