# Supplementary material for: Initial development and validation of a mitochondrial disease quality of life scale
Source: Neuromuscul Disord. 2013 Apr;23(4):324–9. doi: 10.1016/j.nmd.2012.12.012 (PMC3841574; doi:10.1016/j.nmd.2012.12.012)
Supplement: Supplementary Table e-3 — A table illustrating that genotype (one-way analysis of variance) gender (independent t-test) and age (Pearson’s moment correlation co-efficient) have no impact on NMQ domain scores when statistical significance is set at p < 0.003 (Bonferroni correction). [file mmc5.docx]

table e-3

|  | Genotype (one-way analysis of variance) | | Gender (T-test for equality of mean) | | Age (Pearson’s moment correlation coefficient) | |
| --- | --- | --- | --- | --- | --- | --- |
| NMQ Domains | F | p | t | Sig.2-tailed | r | Sig. 2-tailed |
| ADL | 2.47 | 0.02 | -0.453 | 0.681 | 6.136 | 0.120 |
| Mobility | 1.83 | 0.78 | 0.78 | 0.435 | 0.24 | 0.781 |
| Energy Levels/Fatigue | 1.18 | 0.32 | -0.115 | 0.909 | -0.048 | 0.581 |
| Communication | 2.08 | 0.04 | -5.59 | 0.577 | 0.052 | 0.553 |
| Vision/Eyesight | 2.70 | 0.009 | 1.148 | 0.253 | -1.21 | 0.165 |
| Memory/ Cognition | 2.097 | 0.41 | 1.577 | 0.117 | -0.099 | 0.257 |
| Food and digestion | 1.011 | 0.431 | 0.005 | 0.996 | 0.132 | 0.132 |
| Pain | 0.975 | 0.459 | -0.244 | 0.807 | -0.107 | 0.221 |
| Muscle stiffness | 0.96 | 0.467 | -1.065 | 0.289 | -0.174 | 0.046 |
| Migraine/ Headaches | 0.856 | 0.556 | 0.126 | 0.9 | -0.150 | 0.085 |
| Emotional well being | 1.03 | 0.418 | 0.296 | 0.768 | 0.125 | 0.155 |
| Stigma | 1.50 | 0.166 | 0.336 | 0.737 | 0.184 | 0.035 |
| Family role | 0.862 | 0.551 | -1.88 | 0.062 | 0.105 | 0.233 |
| Personal Relationships | 1.694 | 0.106 | -1.122 | 0.264 | 0.097 | 0.269 |
| Social Role/ support | 1.448 | 0.183 | -1.116 | 0.267 | -0.002 | 0.984 |
| Diabetes | 1.35 | 0.226 | 0.022 | 0.983 | 0.250 | 0.004 |
